# Histopathologic Features of Mucosal Head and Neck Cancer Cachexia

**DOI:** 10.1155/2024/5339292

**Published:** 2024-06-27

**Authors:** Alexander J. Jones, Leah J. Novinger, Andrea Bonetto, Kyle P. Davis, Marelle M. Giuliano, Avinash V. Mantravadi, Michael W. Sim, Michael G. Moore, Jessica A. Yesensky

**Affiliations:** ^1^ Indiana University School of Medicine Department of Otolaryngology-Head & Neck Surgery, Indianapolis, IN, USA; ^2^ University of Colorado Anschutz Department of Pathology, Aurora, CO, USA; ^3^ St. Louis University School of Medicine Department of Otolaryngology-Head & Neck Surgery, St. Louis, MO, USA

## Abstract

**Objective:**

Determine the histopathologic features that correlate with head and neck cancer (HNC) cachexia.

**Methods:**

A single-institution, retrospective study was performed on adults with HPV-negative, mucosal squamous cell carcinoma of the aerodigestive tract undergoing resection and free flap reconstruction from 2014 to 2019. Patients with distant metastases were excluded. Demographics, comorbidities, preoperative nutrition, and surgical pathology reports were collected. Comparisons of histopathologic features and cachexia severity were made.

**Results:**

The study included 222 predominantly male (64.9%) patients aged 61.3 ± 11.8 years. Cachexia was identified in 57.2% patients, and 18.5% were severe (≥15% weight loss). No differences in demographics were identified between the groups. Compared to control, patients with severe cachexia had lower serum hemoglobin (*p*=0.048) and albumin (*p* < 0.001), larger tumor diameter (*p* < 0.001), greater depth of invasion (*p* < 0.001), and elevated proportions of pT4 disease (*p* < 0.001), pN2-N3 disease (*p*=0.001), lymphovascular invasion (*p*=0.009), and extranodal extension (*p*=0.014). Multivariate logistic regression identified tumor size (OR [95% CI] = 1.36 [1.08–1.73]), oral cavity tumor (OR [95% CI] = 0.30 [0.11–0.84]), and nodal burden (OR [95% CI] = 1.16 [0.98–1.38]) as significant histopathologic contributors of cancer cachexia.

**Conclusions:**

Larger, more invasive tumors with nodal metastases and aggressive histologic features are associated with greater cachexia severity in mucosal HNC.

## 1. Introduction

Head and neck cancer (HNC) encompasses a diverse group of malignancies and accounted for 54,010 new cases and 10,850 cancer deaths in 2021 [1]. Squamous cell carcinoma (SCC) is the predominant histology usually resulting from significant exposure to tobacco and/or alcohol. Therefore, most tumors occupy the mucosal surface of the upper aerodigestive tract (oral cavity, oropharynx, hypopharynx, and larynx) and cause significant morbidity due to impaired mastication, deglutition, and/or respiration [2].

Advanced HNC is associated with high proportions of unintentional weight loss often despite nutritional supplementation resulting in a catabolic state known as cancer cachexia [2–5]. This is a detrimental condition caused by the interaction between the immune system and malignancy [6]. This interplay releases inflammatory and catabolic mediators which promote skeletal muscle and adipose wasting, fatigue, anorexia, and metabolic derangements [6]. Ultimately, there is an imbalance of energy and unintentional wasting of body mass. As such, it is diagnosed primarily by unintentional weight loss with or without being underweight and sarcopenic [7, 8]. Cancer cachexia unfortunately thus far has limited, if any, improvement with conventional nutrition methods [9, 10]. This comorbidity results in poorer tolerance and outcomes with surgery, chemotherapy, radiation therapy, and survival [11–17].

HNC induces the third highest incidence of cancer cachexia behind pancreatic and gastroesophageal malignancies [6]. It occurs most commonly in advanced tumors of the pharynx and supraglottis, which also have the poorest overall five-year survival within the head and the neck [4]. As an estimated 30% of cancer deaths are related to the consequences of cachexia (e.g., cardiopulmonary decline), a substantial portion of advanced HNC patients will also succumb to cachexia's effect [18]. Despite the preponderance of cachexia in these patients, there is a paucity of investigations into HNC cachexia and limited *in vivo* or *in vitro* models [19, 20]. Therefore, we sought to identify histopathologic features of mucosal HNC which promote cancer cachexia that may provide insight into its pathoetiology. Given the increased incidence of cachexia in more advanced cancers and greater tumor burden [4, 21–23], we hypothesized that larger tumors with more aggressive features would be correlated with worse cachexia severity.

## 2. Patients and Methods

After exemption for review from our Institutional Review Board, a retrospective review was performed of consecutive, adult patients with head and neck mucosal SCC undergoing oncologic ablation and reconstruction at our academic, tertiary referral center from 2014 to 2019. Patients were excluded for any of the following: (1) no pathological report; (2) no primary tumor (Tx/T0 disease); (3) HPV/p16-positive disease; (4) concurrent primary malignancy or distant metastases; (5) lack of or inadequate 30-day preoperative abdominal CT images; (6) active immunosuppression; (7) obvious source of malnutrition (e.g., prior small bowel resection).

Data collected included patient demographics, body mass index (BMI), preoperative 6-month weight loss, skeletal muscle index (SMI), comorbidities (classified as modified Charlson Comorbidity Index, mCCI) [24], performance status (Eastern Cooperative Oncology Group [ECOG] score) [25], prior cancer treatments, primary tumor largest dimension, tumor depth of invasion (DOI), number and largest dimension of positive cervical node, tumor grade, pathologic TNM staging according to the 8^th^ edition of AJCC (American Joint Committee on Cancer) Cancer Staging Manual [26], and presence of lymphovascular invasion (LVI), perineural invasion (PNI), or extranodal extension (ENE). Cancer cachexia was diagnosed according to the international consensus established by Fearon et al. with the presence of active malignancy and unintentional weight loss (1) >5% over the prior 6 months, (2) >2% over prior 6 months with BMI <20 kg/m^2^, and/or (3) weight loss >2% over prior 6 months with sarcopenia [7]. SMI was calculated as previously described using SliceOMatic 5.0 software (TomoVision, Magog, Canada) [27], and sarcopenia was determined when SMI <41.6 cm^2^/m^2^ or <32.0 cm^2^/m^2^ for males and females, respectively [8, 28]. The severity of cancer cachexia was then stratified according to weight loss as mild-moderate (cachexia diagnosis with weight loss ≤14.9%) and severe (weight loss ≥15%).

Statistical analyses were performed using SPSS v28.0 (IBM, Armonk, NY). Continuous and ordinal data reported as mean ± standard deviation or median [range], respectively. Categorical data were presented as number (%). Normality was determined using the Shapiro–Wilk test. Univariate analyses were performed using Welch's *t*-test, Mann–Whitney *U*-test, or Fisher's exact test. Comparisons of multiple groups after stratifying cachexia severity were performed with 2-sided Welch's one-way ANOVA with Tukey post-hoc analysis, Kruskal–Wallis one-way ANOVA with post-hoc analysis, or Pearson's *χ*^2^ test. Significance was determined at *p* < 0.05. With two group comparisons, effect sizes and 95% confidence intervals (95%CI) were measured using Cohen's d for normally distributed continuous data, Goodman and Kruskal's *γ* for ordinal data, and Cramer's v for categorical data. For three or more group comparisons, effect sizes with 95% CI were measured with *ω*^2^ for continuous variables (fixed-effect model) or Cramer's v for categorical variables. All comparisons and effect sizes were determined with bootstrapping using 1000 samples. Binary logistic regression was performed on histopathologic features on the presence of cancer cachexia. Variables with *p* < 0.10 on univariate regression were included in the initial multivariate regression and then subsequently removed using the backward Wald method. Those variables with *p* < 0.10 were included in the final model.

## 3. Results

### 3.1. Overall Summary

A summary of the cohort is displayed in Table 1. A total of *n* = 222 patients were included, of which *n* = 127 (57.2%) met cancer cachexia diagnosis. The majority were white (94.6%) males (64.9%) with mean age and BMI of 61.3 ± 11.8 years and 24.9 ± 6.5 kg/m^2^, respectively. Though most patients reported some difficulty with an oral diet (58.1%), only 26.1% were partially or totally dependent upon tube feeding for nutrition. Radiographic sarcopenia was identified in 27.9% of individuals. The most common comorbidities recorded were hypertension (53.9%) and chronic obstructive pulmonary disease (31.5%). Significant tobacco use (80.2%) and current/prior alcoholism (44.1%) were highly prevalent. Median hemoglobin (Hgb) and serum albumin were 13.1 [7.3–17.2] g/dL and 4.0 [1.5–5.0] g/dL, respectively. Most patients were afflicted with intermediate-grade (71.7%) tumors occupying the oral cavity (69.8%) with average diameter of 4.2 ± 2.0 cm and DOI of 20.1 ± 13.8 mm, resulting in mostly pT4 disease (56.3%). Nearly half (48.6%) of patients had nodal disease, and most of which were classified as pN3 (22.5%), with largest involved node having mean dimensions 2.0 ± 1.3 cm. Nearly one-third of the cohort had prior radiation therapy and/or chemotherapy (CRT).

### 3.2. Cachexia versus Control

A comparison of the cachectic and control groups is listed in Table 1. No difference was detected in demographics, comorbidities, tobacco use, or prior CRT between cachectic and noncachectic groups, although alcohol abuse was more prevalent in cachectic patients (*p* = 0.040, *v* = 0.146). Cachectic patients had lower BMI (*p* < 0.001, *d* = −0.600), more dysphagia and preoperative tube feeding requirements (*p* < 0.001, *v* = 0.368) greater incidence of sarcopenia (*p* = 0.004, *v* = 0.193), and higher distribution of ECOG scores (*p* < 0.001, *γ* = 0.379). They also had slightly lower distributions of preoperative Hgb (*p* = 0.018, *γ* = −0.200) and serum albumin (*p* < 0.001, *γ* = −0.462). Tumors of cachectic individuals less frequently occupied the oral cavity (*p* = 0.001, *v* = 0.231) and were significantly larger in diameter (*p* < 0.001, *d* = 0.716) and DOI (*p* < 0.001, *d* = 0.481), which in turn were more frequently staged pT4 (*p* < 0.001, *v* = 0.284). No differences in tumor grade or incidence of PNI or LVI were observed. Similarly, there were no distinctions of nodal burden, involved nodal size, nodal stage, or ENE. Overall prognostic stage IV disease was higher in the cachectic group (*p* < 0.001, *v* = 0.266).

### 3.3. Histopathologic Features with Cachexia Severity

Comparisons of patient features after stratifying by cachexia severity are listed in Table 2. When cachexia was stratified by severity, there remained no difference in age, sex, comorbidities, substance use histories, or previous CRT. The difference in BMI (*p* < 0.001, *ω*^2^ = 0.088), ECOG (*p* < 0.001, *ω*^2^ = 0.079), preoperative tube feeding (*p* < 0.001, *v*=0.291), and sarcopenia incidence (*p* < 0.003, *v*=0.231) became more drastic with increase in cachexia severity. A small disparity of Hgb was identified in both degrees of cachexia versus noncachectic patients (*p*=0.048, *ω*^2^ = 0.032). Although no variation in serum albumin was seen between cachexia severities, the distribution was lower in both groups compared to control (*p* < 0.001, *ω*^2^ = 0.091). Tumors of the pharynx and larynx were more frequently associated with worse cachexia (*p*=0.002, *v*=0.233). Strong positive associations of tumor size (*p* < 0.001, *ω*^2^ = 0.149), DOI (*p* < 0.001, *ω*^2^ = 0.059), and T4 disease (*p* < 0.001, *v*=0.294) were identified with cachexia progression. Similarly, higher nodal burden (*p*=0.001, *ω*^2^ = 0.053), N-stage (*p*=0.001, *v*=0.246), and status of ENE (*p*=0.014, *v*=0.196) were identified with higher degrees of cachexia. Although no discrepancy was noted with prevalence of PNI, there was a significant increase in incidence of LVI between severe cachexia and control or mild-moderate cachexia (75.6% vs. 55.8% and 46.5%, respectively; *p*=0.009, *v*=0.207). Overall, these factors contributed to increased proportion of stage IV aerodigestive SCC with greater cachexia burden (*p* < 0.001, *v*=0.285).

### 3.4. Spearman Correlations

Spearman correlations (*ρ*) of weight loss, BMI, and different disease-related continuous variables are demonstrated in Table 3. Moderate strength relationships were observed between weight loss and tumor size (*ρ* = −0.461, *p* < 0.001), tumor DOI (*ρ* = −0.321, *p* < 0.001), and serum albumin (*ρ* = −0.310, *p* < 0.001), while weak associations were seen with nodal disease (*ρ* = 0.222, *p* < 0.001) and Hgb (*ρ* = −0.197, *p*=0.005). BMI, aside from serum albumin (*ρ* = 0.165, *p*=0.042), failed to demonstrate any significant association between tumor size, tumor DOI, nodal disease, or Hgb. Both tumor size and DOI had weak associations with Hgb (*ρ* = −0.287 and −0.293, respectively; *p* < 0.001) and serum albumin (*ρ* = −0.268 and −0.252, respectively; *p* < 0.001), but nodal burden had no significant correlation to Hgb or albumin. Expectedly, both tumor size and DOI were well correlated to nodal burden and size.

### 3.5. Logistic Regression of Histopathologic Features on Cancer Cachexia

Univariate and multivariate regressions of the histopathologic features upon cancer cachexia status are displayed in Table 4. Variables with *p* < 0.10 on univariate regression included tumor size, tumor DOI, tumor located other than oral cavity, nodal burden, largest node, and PNI. After stepwise backward regression, the final model included tumor size (*p*=0.011, OR [95% CI] = 1.36 [1.08–1.73]), oral cavity tumor (*p*=0.023, OR [95%CI] = 0.30 [0.11–0.84]), and nodal burden (*p*=0.090, OR [95% CI] = 1.16 [0.98–1.38]).

## 4. Discussion

This investigation reports the first review of histopathologic features of nonmetastatic mucosal head and neck SCC which are correlated with cancer cachexia and its severity. We demonstrate the effect of tumor size, invasion, nodal disease, grade, and adverse features on cachexia severity and their associations with the clinical criteria for cachexia diagnosis. This lays important groundwork into undermining the pathoetiology of cancer cachexia for this population of patients in whom cachexia is highly prevalent and detrimental to prognosis.

The underlying mechanism of cancer cachexia is complex but originates in the tumor-host interaction. There is first the tumor “secretome” – procatabolic molecules (e.g., TGF-*β*, heat shock proteins) produced from the tumor itself. Second to arise are the inflammatory cytokines and mediators (e.g., IL-1, IFN-*γ*, and TNF-*α*) which are induced by the tumor and host immune system interaction. Together, a widespread inflammatory state is generated and incites skeletal muscle degradation, lipolysis and energy wasting, and central nervous system changes which promote anorexia and fatigue. The target organs of these mediators demonstrate reciprocal interaction (i.e., “crosstalk”) to perpetuate the cachectic response even further [6]. Emerging evidence suggests that there are secondary and tertiary effects of these interactions including bone degradation and altered gut biome [29, 30]. Furthermore, the chemotherapeutic agents used to eradicate the cachexia-inducing tumor can result in similar alterations in homeostasis and propagate its progression [31]. Investigating the tumor-host and multiorgan interactions therefore is imperative for development of targeted cachexia therapeutics.

Tumor size and DOI were strongly associated with cachexia severity in our investigation. Although it is known that larger tumors have greater incidence of cancer cachexia within head and neck cancer [4] as well as several other cancer pathologies [21, 22], the relationship between DOI and cachexia has not previously been examined. DOI is an important histopathological feature that determines tumor classification and overall prognostic staging for carcinoma of the oral cavity [26, 32, 33]. This is similar to cutaneous carcinomas and melanomas where depth of invasion portends worse prognosis than tumor size [26]. In the hypopharynx and larynx, although not included in TNM staging, DOI also predicts nodal disease and disease-free survival [34–36]. Though there is concordance between tumor size and DOI, it is not perfect as tumors can be large and exophytic or small and endophytic and therefore represent separate entities.

In a similar manner, higher tumor burden within local cervical node metastases was identified at the greatest degree of cachexia severity, both number of involved regional nodes, and identification of ENE. Together these correlated to greater pathologic nodal classification in severely cachectic patients. As expected, nodal disease is more frequently identified in larger, greater T-classification primary tumors. With both tumor and nodal classifications combined, the incidence of cancer cachexia increased as staging increased, similar to prior analyses [23]. It is unclear if nodal metastasis in head and neck SCC has a unique role in the cachectic process compared to the primary tumor or is simply an increase in total disease burden. However, evidence suggests that the development of cachexia is linked to the molecular events during progression of low-stage to high-stage disease, advancing from localized tumor to nodal and finally distant metastases [37, 38]. The genetic changes and resulting molecular expression which allow for the malignant propagation also have corresponding local and distant tissue effects.

Given the importance of tumor-host interplay in cancer cachexia, we speculate increased tumor size, tumor DOI, and greater nodal burden all increase the total surface area for such interactions. This may, in turn, enable more opportunities for the cachectic inflammatory response to occur and subsequent systemic cachectic effects. Greater tumor burden in both primary and nodal disease also increases the total tumor cachexia “secretome” and production of tumor-derived cachexia mediators. The signaling and molecular changes that occur with each stage of tumor progression may also coincide with these changes in histopathology, but further investigations are required to confirm or deny these suspicions.

What cannot be explained precisely are the differences between patients with large, invasive and/or regionally-metastatic SCC without any evidence of cachexia versus those with small, localized tumors experiencing significant weight loss and atrophy. This may be due to polymorphisms in cachexia-modulating cytokines and receptors produced by both the tumor and the host. The unique tumor molecular alterations combined with unique patient resistance or susceptibilities can increase or decrease the rate at which cachexia develops, respectively [21]. For instance, hundreds of single nucleotide polymorphisms have been identified in the expression of inflammatory cytokines, proteins, and hormones in several different cancers inducing cachexia [21, 39]. Similar variations in host cytokine receptors can also account for the differences seen in tumor response. In congruence to individual genetic expression differences is the variation in autophagy of the tumor microenvironment which modulates the tumor-immune inflammatory response. Furthermore, the “tumor purity,” proportion of tumor to immune cell infiltrate, fluctuates by cancer histology, and the corresponding host infiltrating immune phenotype has contributed to the heterogeneous cachexia response [40]. All these variations may be akin to the variable inflammatory response and symptoms produced by an illness or effectiveness of a therapeutic drug from one individual to the next. Further analyses are required to determine those factors which promote cachexia progression, potentially through single-cell DNA testing of tumors, host immune cells, skeletal muscle, adipose, etc.

LVI and PNI, although adverse features which have demonstrated worse survival and locoregional control, do not have a role in TNM staging in mucosal HNSCC [26, 41, 42]. Despite this, the presence of either on histopathology generally necessitates adjuvant radio- and/or chemotherapy after surgical resection [41, 42]. PNI occurs when tumor cells are identified within any of the three peripheral nerve sheath layers and allows microscopic regional spread. LVI is identified when tumor aggregates within the walls or endothelium of lymphatic vessels and, similar to PNI, provides a highway for metastasis [42, 43]. Both features had similar prevalence between cachectic patients and controls, but there was a significant increase in LVI prevalence when stratifying by cachexia severity (>70%). This may represent only a correlative incidence with the larger, more invasive tumor and be part of the bigger picture of advanced disease, particularly nodal disease [44]. However, similar mechanisms which contribute to tumor invasion in the epithelial-mesenchymal transition, as seen with DOI and tumor size, are also identified in PNI and LVI. These processes require reciprocal interaction of Schwann cells and lymphatic endothelial cells, which can induce inflammatory cytokine release [41, 44]. Although PNI can induce significant neuropathic pain, limit food intake, and promote malnutrition particularly in oral cavity SCC [41], it was not correlated with greater weight loss in this investigation. A lack of correlation in cancer cachexia and PNI in pancreatic adenocarcinoma has similarly been demonstrated [45].

Tumor grade, a classification of cell atypia, was not associated with cancer cachexia within this study, and it, too, is not included in the AJCC staging of head and neck SCC [26]. Yet, tumor grade has significant prognosis in locoregional and overall survival in these patients [46]. Furthermore, higher levels of atypia in mucosal SCC are correlated with other adverse features including nodal disease, ENE, LVI, and need for postoperative chemotherapy and radiation therapy [46]. Tumor grade remains a critical component of staging in several other head and neck malignancies, such as soft tissue sarcomas and cutaneous carcinomas, and may contribute to the development of cachexia in certain cancers [26]. However, the molecular alterations which occur at varying levels of tumor grade and the resultant tumor-immune interactions and release inflammatory mediators of cachexia are still unknown.

This investigation is limited in its retrospective nature which only included aerodigestive, non-HPV SCC of the head and neck. Although these are the most common head and neck malignancies, it excludes other pathologies such as salivary gland, cutaneous SCC, and sarcomas. Alongside this, there were no serologic biomarkers of inflammation (IL-2, CRP, etc.) with which further correlations could be made. With this and the high rates of dysphagia and prior chemoradiation therapy, we are unable to fully parse out the malnutrition component of the weight loss identified in our HNC patients, though the majority patients with dysphagia and aspiration were initiated on tube-feeding regimens many weeks prior to surgery. Finally, the cohort size is limited due to the requirement of abdominal CT imaging for the measurement of SMI, which are generally obtained only for metastatic workup and/or vascular imaging for fibular free flap reconstruction.

## 5. Conclusions

Mucosal HNC cachexia is associated with larger, more invasive tumors with more adverse features. The molecular role these features have in the development of cancer cachexia requires further investigation.

## Figures and Tables

**Table 1:** Overall summary and comparison of cachectic versus control patients.

Variable	Overall (*n* = 222)	Control (*n* = 95)	Cachexia (*n* = 127)	*P*	Effect size (95% CI)
Age (y)	61.3 ± 11.8	62.5 ± 11.7	61.5 ± 11.9	0.547^a^	−0.082 (−0.347–0.184)
Sex (m)	144 (64.9)	55 (57.9)	89 (70.1)	0.066^b^	0.126 (0.010–0.253)
Race (white)	210 (94.6)	92 (96.8)	118 (92.9)	0.242^b^	0.086 (0.004–0.193)
BMI (kg/m^2^)	24.9 ± 6.5	27.0 ± 6.4	23.2 ± 6.2	<0.001^a^	−0.600 (−0.871–−0.328)
Nutritional intake				<0.001^c^	0.368 (0.256–0.490)
Normal PO	93 (41.9)	59 (62.1)	34 (26.8)		
PO + dysphagia	71 (32.0)	24 (25.3)	47 (37.0)		
Tube feeding	58 (26.1)	12 (12.6)	46 (36.2)		
Comorbidities					
mCCI	1.0 [0-6]	1.0 [0-6]	1.0 [0-5]	0.946^d^	0.007 (−0.204–0.211)
Sarcopenia	62 (27.9)	17 (17.9)	45 (35.4)	0.004^b^	0.193 (0.076–0.314)
ECOG score	1 [0-4]	0 [0-3]	1 [0-4]	<0.001^d^	0.379 (0.175–0.564)
Substance use					
Tobacco	178 (80.2)	75 (78.9)	103 (81.1)	0.735^b^	0.027 (0.044–0.161)
Alcohol	98 (44.1)	34 (35.8)	64 (50.4)	0.040^b^	0.146 (0.022–0.267)
Laboratory values^†^					
Hgb (g/dL)	13.1 [7.3-17.2]	13.4 [8.0-17.2]	12.6 [7.3-16.5]	0.018^d^	−0.200 (−0.359–−0.033)
Albumin (g/dL)	4.0 [1.5-5.0]	4.2 [1.5-4.7]	3.9 [2.4-5.0]	<0.001^d^	−0.462 (−0.622–−0.296)
Primary tumor					
Location					
Oral cavity	155 (69.8)	78 (82.1)	77 (60.6)	<0.001^b^	0.231 (0.121–0.342)
OP/HP	29 (13.1)	7 (7.4)	22 (17.3)	0.043^b^	0.146 (0.028–0.263)
Larynx	39 (17.6)	11 (11.6)	28 (22.0)	0.050^b^	0.136 (0.019–0.259)
Size (cm)	4.2 ± 2.0	3.5 ± 1.7	4.8 ± 2.0	<0.001^a^	0.716 (0.441–0.989)
DOI (mm)	20.1 ± 13.8	16.4 ± 13.2	22.9 ± 13.7	<0.001^a^	0.481 (0.20–0.755)
Grade^†^				0.324^d^	−0.143 (−0.429–0.123)
Low (I)	12 (5.5)	3 (3.2)	9 (7.1)		
Intermediate (II)	157 (71.7)	67 (72.0)	90 (71.4)		
High (III)	50 (22.8)	23 (24.7)	27 (21.4)		
T-stage				<0.001^b^	0.284 (0.152–0.408)
T1-T3	97 (43.7)	57 (60.0)	40 (31.5)		
T4	125 (56.3)	38 (40.0)	87 (68.5)		
Nodal disease					
Total	0 [0-15]	0 [0-7]	1 [0-15]	0.087^d^	0.186 (−0.020–0.369)
Largest node (cm)^‡^	2.0 ± 1.3	1.7 ± 1.1	2.1 ± 1.4	0.054^a^	0.347 (−0.029–0.722)
N-Stage				0.075^b^	0.121 (0.013–0.258)
N0-N1	132 (59.5)	63 (66.3)	69 (54.3)		
N2-N3	90 (40.5)	32 (33.7)	58 (45.7)		
Adverse features					
PNI	131 (59.0)	50 (52.6)	81 (63.8)	0.100^b^	0.112 (0.010–0.246)
LVI	124 (55.9)	53 (55.8)	71 (55.9)	>0.99^b^	0.001 (<0.001 – 0.146)
ENE	62 (27.9)	22 (23.2)	40 (31.5)	0.178^b^	0.092 (0.007–0.211)
Overall stage				<0.001^b^	0.266 (0.135–0.399)
I–III	71 (32.0)	44 (46.3)	27 (21.3)		
IV	151 (68.0)	51 (53.7)	100 (78.7)		
Previous treatment	69 (31.1)	27 (28.4)	42 (33.1)	0.469^b^	0.050 (0.002–0.181)
Radiation therapy	62 (27.9)	26 (27.4)	36 (28.3)	>0.99^b^	0.011 (0.001–0.144)
Chemotherapy	50 (22.5)	17 (17.9)	33 (26.0)	0.194^b^	0.096 (0.005–0.223)

Data listed as mean ± standard deviation for normally distributed continuous data, median [range] for non-normally distributed continuous or ordinal data, and number (%) for categorical data. Effect sizes listed as Cohen's d for normally distributed continuous data, Goodman and Kruskal's *γ* for ordinal data, and Cramer's v for categorical data. Abbreviations: BMI, body mass index; CI, confidence interval; DOI, depth of invasion; ECOG, Eastern Cooperative Oncology Group; ENE, extranodal extension; Hgb, hemoglobin; LVI, lymphovascular invasion; mCCI, modified Charlson Comorbidity Index; OP/HP, oropharynx/hypopharynx; PNI, perineural invasion; PO, per oral diet. ^†^Less than total amount of available patients with data recorded: Hgb, *n* = 198; albumin, *n* = 153; tumor grade, *n* = 219. ^‡^Only for *n* = 117 patients with nodal disease and dimension reported for analysis. ^a^Welch's *t*-test, 2 -tailed; ^b^Fisher's exact test, 2-tailed; ^c^Pearson's *χ*^2^ test, 2-tailed; ^d^Mann–Whitney *U* test, 2-tailed.

**Table 2:** Comparison of patient and histopathologic features by cancer cachexia severity.

Variable	Control (*n* = 95)	Mild-moderate cachexia (*n* = 86)	Severe cachexia (*n* = 41)	*P*	Effect size (95%CI)
Age (y)	62.5 ± 11.7	62.3 ± 12.3	59.8 ± 10.9	0.396^a^	−0.002 (−0.009–0.030)
Sex (m)	55 (57.9)	61 (70.9)	28 (68.3)	0.163^b^	0.128 (0.035–0.267)
Race (white)	92 (69.8)	77 (89.5)	41 (100)	NA	0.185 (0.094–0.296)
BMI (kg/m^2^)	27.0 ± 6.4	24.0 ± 6.1^dd^	21.7 ± 6.1^ddd^	<0.001^a^	0.088 (0.022–0.162)
Nutritional intake				<0.001^b^	0.291 (0.224–0.376)
Normal PO	59 (62.1)	30 (34.9)	4 (9.8)		
PO + dysphagia	24 (25.3)	27 (31.4)	20 (48.8)		
Tube feeding	12 (12.6)	29 (33.7)	17 (41.5)		
Comorbidities					
mCCI	1.0 [0-6]	1.0 [0-5]	1.0 [0-3]	0.985^c^	−0.008 (−0.009–0.002)
Sarcopenia	17 (17.9)	26 (30.2)	19 (46.3)	0.003^b^	0.231 (0.113–0.367)
ECOG score	0 [0-3]	1 [0-4]^f^	2 [0-3]^fffg^	<0.001^c^	0.079 (0.017–0.151)
Substance use					
Tobacco	75 (78.9)	68 (79.1)	35 (85.4)	0.653^b^	0.062 (0.018–0.204)
Alcohol	34 (35.8)	42 (48.8)	22 (53.7)	0.084^b^	0.150 (0.044–0.287)
Laboratory values^†^					
Hgb (g/dL)	12.7 [8.3-16.5]	12.6 [8.4-15.8]^f^	13.0 [7.3-16.0]^f^	0.048^c^	0.032 (0.009–0.093)
Albumin (g/dL)	4.2 [1.5-4.7]	3.9 [2.5-5.0]^fff^	3.9 [2.4-4.8]^fff^	<0.001^c^	0.091 (0.013–0.183)
Primary tumor					
Location					
Oral cavity	78 (82.1)	53 (61.6)	24 (58.5)	0.002^b^	0.233 (0.127–0.360)
OP/HP	7 (7.4)	14 (16.3)	8 (19.5)	0.082^b^	0.150 (0.051–0.289)
Larynx	11 (11.6)	18 (20.9)	10 (24.4)	0.114^b^	0.140 (0.042–0.278)
Size (cm)	3.5 ± 1.7	4.4 ± 1.9^dd^	5.6 ± 1.9^dddee^	<0.001^a^	0.149 (0.066–0.232)
DOI (mm)	16.4 ± 13.2	21.4 ± 13.0^d^	26.1 ± 14.6^ddd^	<0.001^a^	0.059 (0.005–0.128)
Grade				0.614^c^	−0.004 (−0.009–0.023)
Low (I)	3 (3.2)	7 (8.2)	2 (4.9)		
Intermediate (II)	67 (72.0)	59 (69.4)	31 (75.6)		
High (III)	23 (24.7)	19 (22.4)	8 (19.5)		
T-stage				<0.001^b^	0.294 (0.180–0.417)
T1–T3	57 (60.0)	30 (34.9)	10 (24.4)		
T4	38 (40.0)	56 (65.1)	31 (75.6)		
Nodal disease^‡^					
Total	0 [0-7]	0 [0-15]	2 [0-13]^fffggg^	0.001^c^	0.053 (0.002–0.119)
Largest node (cm)	1.7 ± 1.1	2.0 ± 1.5	2.4 ± 1.3	0.059^a^	0.025 (−0.017–0.106)
N-Stage				0.001^b^	0.246 (0.126–0.394)
N0-N1	63 (66.3)	55 (64.0)	14 (34.1)		
N2-N3	32 (33.7)	31 (36.0)	27 (65.9)		
Adverse features					
PNI	50 (52.6)	52 (60.5)	29 (70.7)	0.135^b^	0.134 (0.034–0.278)
LVI	53 (55.8)	40 (46.5)	31 (75.6)	0.009^b^	0.207 (0.102–0.334)
ENE^‡^	22 (23.2)	21 (24.4)	19 (46.3)	0.014^b^	0.196 (0.075–0.357)
Overall stage				<0.001^b^	0.285 (0.178–0.407)
I–III	44 (46.3)	22 (25.6)	5 (12.2)		
IV	51 (53.7)	64 (74.4)	36 (87.8)		
Previous treatment	27 (28.4)	33 (38.4)	9 (22.0)	0.132^b^	0.135 (0.047–0.271)
Radiation therapy	26 (27.4)	28 (32.6)	8 (19.5)	0.305^b^	0.103 (0.032–0.237)
Chemotherapy	17 (17.9)	25 (29.1)	8 (19.5)	0.175^b^	0.125 (0.037–0.267)

Data listed as mean ± standard deviation for normally distributed continuous data, median [range] for non-normally distributed continuous or ordinal data, and number (%) for categorical data. Effect sizes written as *ω*^2^ for continuous and ordinal variables (fixed-effect model) or Cramer's v for categorical variables. Mild-moderate cachexia determined with any cachexia diagnosis and <15% weight loss, while severe cachexia determined at ≥15% weight loss. Abbreviations: BMI, body mass index; DOI, depth of invasion; ECOG, Eastern Cooperative Oncology Group; ENE, extranodal extension; Hgb, hemoglobin; LVI, lymphovascular invasion; mCCI, modified Charlson Comorbidity Index; OP/HP, oropharynx/hypopharynx; PNI, perineural invasion; PO, per oral diet. ^†^Less than total amount of available patients with data recorded: Hgb, *n* = 198; albumin, *n* = 153. ^‡^Only for *n* = 117 patients with nodal disease and dimension reported for analysis. ^a^One-way Welch's ANOVA with Tukey's post-hoc analysis, 2 -tailed; ^b^Pearson's *χ*^2^ analysis, 2-tailed; ^c^Kruskal–Wallis one-way ANOVA, 2-tailed. Tukey's post-hoc analyses demonstrating ^d^*p* < 0.05 versus control; ^dd^*p* < 0.01 versus control; ^ddd^*p* < 0.001 versus control; ^e^*p* < 0.05 versus mild-moderate cachexia; ^ee^*p* < 0.01 versus mild-moderate cachexia; ^eee^*p* < 0.001 versus mild-moderate cachexia. Kruskal–Wallis post-hoc analyses demonstrating ^f^*p* < 0.05 versus control; ^ff^*p* < 0.01 versus control; ^fff^*p* < 0.001 versus control; ^g^*p* < 0.05 versus mild-moderate cachexia; ^gg^*p* < 0.01 versus mild-moderate cachexia; ^ggg^*p* < 0.001 versus mild-moderate cachexia.

**Table 3:** Correlations of continuous variables and features of cancer cachexia.

	BMI	WL (%)	Tumor size	Tumor DOI	Total nodes	Largest node	Hgb	Albumin
BMI								
WL (%)	−0.291^‡^							
Tumor size	−0.051	0.461^‡^						
Tumor DOI	−0.002	0.321^‡^	0.727^‡^					
Total nodes	−0.069	0.222^‡^	0.347^‡^	0.246^‡^				
Largest node	−0.097	0.156	0.353^‡^	0.294^‡^	0.456^‡^			
Hgb	0.113	−0.197^†^	−0.287^‡^	−0.293^‡^	−0.032	−0.164		
Albumin	0.165^*∗*^	−0.378^‡^	−0.268^‡^	−0.252^‡^	−0.077	−0.178	0.454^‡^	

Spearman correlations (*ρ*) are listed for continuous variables. Weak associations demonstrated with 0.1 ≤ |*ρ*| < 0.3, moderate associations with 0.3 ≤ |*ρ*| < 0.5, and strong associations with |*ρ*| ≥ 0.5. ^*∗*^*p* < 0.05, ^†^*p* < 0.01, ^‡^*p* < 0.001. Abbreviations: BMI, body mass index; DOI, depth of invasion; Hgb, hemoglobin; WL, 6-month weight loss.

**Table 4:** Binary logistic regression of histopathologic variables on cancer cachexia.

Variable	Univariate		Multivariate	
	*P*	OR (95% CI)	*P*	OR (95% CI)
Tumor size (cm)	<0.001	1.54 (1.29–1.84)	0.011	1.36 (1.08–1.73)
Tumor DOI (mm)	<0.001	1.04 (1.02–1.06)		
Tumor location (oral cavity)	<0.001	0.34 (0.18–0.63)	0.023	0.30 (0.11–0.84)
Tumor grade (cont.)	0.295	0.75 (0.44–1.28)		
Total nodes	0.010	1.18 (1.04–1.34)	0.090	1.16 (0.98–1.38)
Largest node (cm)	0.075	1.34 (0.97–1.84)		
PNI	0.096	1.59 (0.92–2.72)		
LVI	0.986	1.01 (0.59–1.72)		
ENE	0.172	1.53 (0.83–2.80)		
Prior CRT	0.459	1.24 (0.70–2.22)		

Variables with *p* < 0.10 on univariate regression were included in the initial multivariate regression model (tumor size, tumor DOI, oral cavity, nodal burden, total nodes, PNI). Variables were removed sequentially in the backward Wald method with all final variables having *p* < 0.10. CI, confidence interval; CRT, chemoradiation therapy; DOI, depth of invasion; ENE, extranodal extension; LVI, lymphovascular invasion; OR, odds ratio; PNI, perineural invasion.

## Data Availability

The data that support the findings of this study are available on request from the corresponding author. The data are not publicly available due to privacy or ethical restrictions.
